# Cardiovascular risk factors and carotid intima-media thickness with neurocognitive dysfunction in people living with HIV on stable combination anti-retroviral therapy

**DOI:** 10.1007/s13205-023-03865-9

**Published:** 2024-02-15

**Authors:** Unnathi Nayak, Nikhil Victor Dsouza, P. V. Santosh Rai, Basavaprabhu Achappa, Ramesh Holla, B. V. Murlimanju

**Affiliations:** 1 Intern, Kasturba Medical College, Mangalore, Manipal Academy of Higher Education, Manipal, India; 2 Department of Internal Medicine, Kasturba Medical College, Mangalore, Manipal Academy of Higher Education, Manipal, India; 3 Department of Radiodiagnosis, Kasturba Medical College, Mangalore, Manipal Academy of Higher Education, Manipal, India; 4 Department of Community Medicine, Kasturba Medical College, Mangalore, Manipal Academy of Higher Education, Manipal, India; 5 Department of Anatomy, Kasturba Medical College, Mangalore, Manipal Academy of Higher Education, Manipal, India

**Keywords:** Carotid intima-media thickness, Cardiovascular risk factors, Cognitive dysfunction, Montreal cognitive assessment, Anti-retroviral agents

## Abstract

The goal of this clinical research was to determine the relationship between carotid intima-media thickness (cIMT), cardiovascular risk factors, and neuro-cognitive function in people living with HIV (PLHIV) and were on stable combination anti-retroviral therapy (cART). This is a cross-sectional study performed at a single center, including 149 patients who visited the anti-retroviral therapy center of our tertiary care hospital. Among the PLHIV of our research, 62.4% had at least one associated cardiovascular risk factor, and 61.1% of them had abnormally high cIMT (≥ 0.9 mm on any one side, *p* = 0.035). These factors and being the male gender (*p* = 0.028) were associated with a greater Framingham 10-year risk percentage. Hypercholesterolemia was observed in 30.9% of the PLHIV and a higher body mass index (≥ 25 kg/m^2^) was found in 26.8% of them. The cognitive impairment was milder in 71.8% of cases and moderate in 9.4% of PLHIV. The Chi-square test revealed that a higher proportion of participants who had lower HDL-C levels (*p* = 0.045), smokers (*p* = 0.029), systolic blood pressure ≥ 140 mmHg (*p* = 0.012), and lower educational status (*p* = 0.017) had a poorer cognitive performance. In our sample population, a higher prevalence of elevated cIMT, cardiovascular risk factors, and mild and moderate cognitive deficiency was observed in PLHIV, who were on stable cART. However, routine assessment of the neuropsychological functions and management of modifiable risk factors are not performed in our patients. Further exploration of the relationship between cardiovascular risks, cIMT, and cognitive impairment in PLHIV is essential to formulate the guidelines and delay the onset of neurocognitive disorders in these patients.

## Introduction

As per the data, by the end of 2020, 37.7 million people are living with human immunodeficiency virus (PLHIV) infection and HIV-related illness (Dzinamarira et al. 2022). Advent of combination anti-retroviral therapy (cART) has significantly enriched the best clinical outcome and survival of PLHIV across the world against the 20-year reduction of life expectancy in HIV-AIDS (acquired immunodeficiency syndrome) previously (May et al. 2014). With effective viral suppression, patients can avoid AIDS-defining illness and delay death. As the PLHIV enters adulthood and attain old age, cardiovascular ailments and co-morbidities like hypertension, diabetes, and dyslipidemia become clinically significant, leading to the deterioration of vascular health (Palella et al 2006; Bijker et al. 2019). With aging and effective viral suppression, vascular and metabolic factors gain importance over conventional HIV-related risk factors in predicting poor cognitive performance (Ciccarelli et al. 2015). Age, diabetes, hyperlipidemia, systolic hypertension, obesity, previous cardiovascular ailment, and carotid intima-media thickness are found to be linked to cognitive impairment in PLHIV with well-suppressed viral replication (Simioni et al. 2010). ART drugs themselves can cause neurotoxicity, and protease inhibitors in particular, are known to cause lipodystrophy, hyperlipidemia, and insulin resistance (Ciccarelli et al. 2015).

As a consequence, atherosclerosis of carotid arteries and cerebral vessels leads to circulatory disturbance and chronic hypoperfusion with subsequent impaired cognitive function. Carotid intima-media thickness (cIMT) is an effective tool for detecting subclinical atherosclerosis and predicting future risk for cerebral and myocardial infarction. In PLHIV, higher cIMT associated with a higher Framingham Risk score and lower overall cognitive performance has been described. However, risk factors like diabetes can act as confounders and cIMT may no longer be an independent prognosticator (Ances et al. 2009; Jericó et al. 2006).

In PLHIV, who receive cART, cardiovascular disease is independently linked to poor cognitive outcomes as in the general population. Cardiovascular co-morbidity is attributed to HIV-associated neurocognitive disorders (HAND) pathogenesis, which is more important than the management of HIV infection itself (Moulignier and Costagliola 2020). Though the mechanism of cognition decline and aging of the brain in PLHIV is not well-understood, it may be because of the inflammation of the brain and cerebrovascular changes. It is reported that poorly managed cardiovascular disease and HIV can lead to brain damage. Hence, it is advisable to do the early detection and management of cardiovascular co-morbidity (Moulignier and Costagliola 2020).

HAND, like AIDS-associated dementia and lymphomas of the central nervous system, proved to be higher in the pre-ART era. AIDS is a state of chronic inflammation and immune activation with direct effects on the brain and indirect mechanisms causing HAND. High viral load at diagnosis, high plasma viral loads, and duration of infection were associated with poor cognition. While there is a substantial decline in severe neurocognitive impairment after the widespread use of cART, however, HIV-related cognitive diminution still prevails in a milder or asymptomatic form (Simioni et al. 2010; Heaton et al. 2012). Perhaps, other neurodegenerative, metabolic, and vascular factors combine to accelerate cognitive deficiency in chronic HIV infection (Smail and Brew 2018). As a result, poor execution of daily life activities increases functional dependency and reduces the quality of life, which remains unresolved (Tozzi et al. 2004). There were no research studies available in the Indian population about the cIMT, cardiovascular risk factors, and its association with neurocognitive performance. This study aimed to observe the cardiovascular risk factors, carotid intima-media thickness and its association with neurocognitive dysfunction in PLHIV. The objectives were to measure the carotid intima-media thickness in PLHIV on stable cART, to identify cardiovascular risk factors in PLHIV on stable cART, and to determine the association between carotid intima-media thickness, cardiovascular risk factors, and neuro-cognitive function in PLHIV on stable cART.

## Materials and methods

This present research was conducted in accordance with STROCSS criteria. Approval for this study was obtained from the ethics committee of our institution. This is a cross-sectional study performed at the ART center of our tertiary care medical institution and the PLHIV were studied at this single center. The participants of this study were selected based on non-probability sampling. They included asymptomatic PLHIV in the age group of 18–75 years, as per the criteria by Waldrop et al. (2016). These patients were on ART for at least a year and had no adverse drug reactions, which required regular monitoring. There should be no history of any chronic illness and ongoing pregnancy (Waldrop et al. 2016). The patients should have a good understanding of lifelong adherence and the evidence of success in the treatment they received. This comprised two consecutive undetectable viral loads and, rise in the CD4 counts above 200 cells/μL. If the viral loads are not available, an objective measurement of adherence was done (Waldrop et al. 2016).

**Fig. 1:**
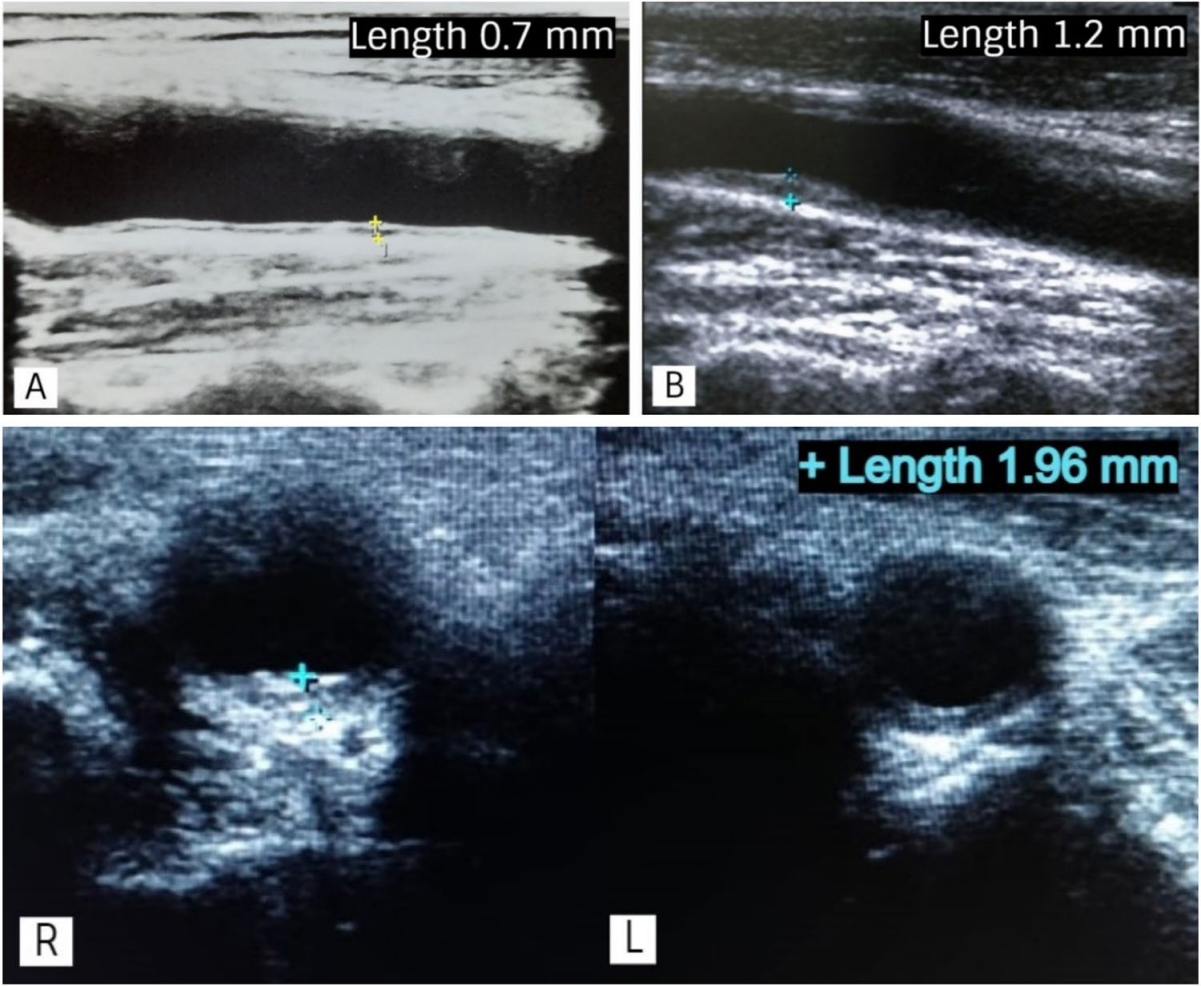
**A** B-mode ultrasound image (longitudinal section) showing the normal measurement (< 0.9 mm) of carotid intima-media thickness; **B** increased thickness (> 0.9 mm); **C** measurement of the carotid intima-media thickness on the right side (axial section); **D** increased thickness (> 0.9 mm) over the left side (axial section)

The patients visiting the ART center were approached on an out-patient basis and engaged in this research after obtaining a signed informed consensus. The exclusion criteria included the patients, who were on statins and antiplatelets, currently hospitalized, being terminally ill, and those who refused to give the consent (Waldrop et al. 2016). Patient’s demographic details and clinical history, including HIV related history, were collected using a structured proforma. Cardiovascular risk factors were evaluated by Framingham 10-year risk scores (National Cholesterol Education Program (NCEP) Expert Panel on Detection, Evaluation, and Treatment of High Blood Cholesterol in Adults, Adult Treatment Panel III, 2002), cIMT by Doppler B scan, neurocognitive assessment by Montreal Cognitive Assessment (MoCA) (Nasreddine et al. 2005) and grooved pegboard test (Merker and Podell 2011). The carotid intima-media thickness (cIMT), defined as ‘the distance between the media‐adventitia interface and the lumen‐intima interface’, was calculated by a single radiologist, who is the co-author of this study. This was blinded to the results of neurocognitive testing and measured by B-mode ultrasonography (GE Logiq S8 expert, GE Healthcare, USA). Both the carotids were assessed by positioning the patient supine, head in the midline, slightly slanted upwards. Three distinct thicknesses were measured 1 cm proximal to the carotid bifurcation, and a mean value calculated on the left or right side ≥ 0.9 mm was considered abnormal (Fig. 1). A MoCA score of 26 or higher was classified normal, 18–25 as mild, 10–17 as moderate, and < 10 as severe cognitive impairments. The grooved pegboard test examines the motor speed, coordination, and generalized neurocognitive slowdown. The mean scores were calculated using the time taken to fill the pegboard on three trials, to take care of the practice effect. Time taken > 100 s was classified as mild impairment and > 300 s represented severe impairment. The patient’s laboratory reports, such as the lipid profile, were traced using the hospital records.

The sample size of 149 in this study was calculated using a power of 80%, confidence level of 95%, relative precision of 8% and prevalence of neurocognitive dysfunction to 52% (Fabbiani et al. 2013). The following formula was considered

$$n = \frac{4PQ}{{D^{2} }}\;({\text{where}}\,p\, = \,{52}\% ,q\, = \,{1} - p\, = \,{48}\% ,d\, = \,{8})$$

The data analysis was performed by the SPSS (Version 25.0 Armonk, NY: IBM Corp). Association of cardiovascular risk factors and Framingham risk score with neurocognitive impairment categories (MoCA, grooved pegboard test), cIMT scores were analyzed using Chi-square test or Fisher’s exact test. The statistically significant variables were carried forward for multivariate analysis using the ordinal and binary logistic regression analysis. Data are given as mean and standard deviation (SD), median, and interquartile range (IQR). A two-tailed ‘*p*’ value of < 0.05 was considered as statistically noteworthy. The variables which underwent logistic regression were presented as adjusted odds ratios with confidence interval and *p *value.

**Fig. 2:**
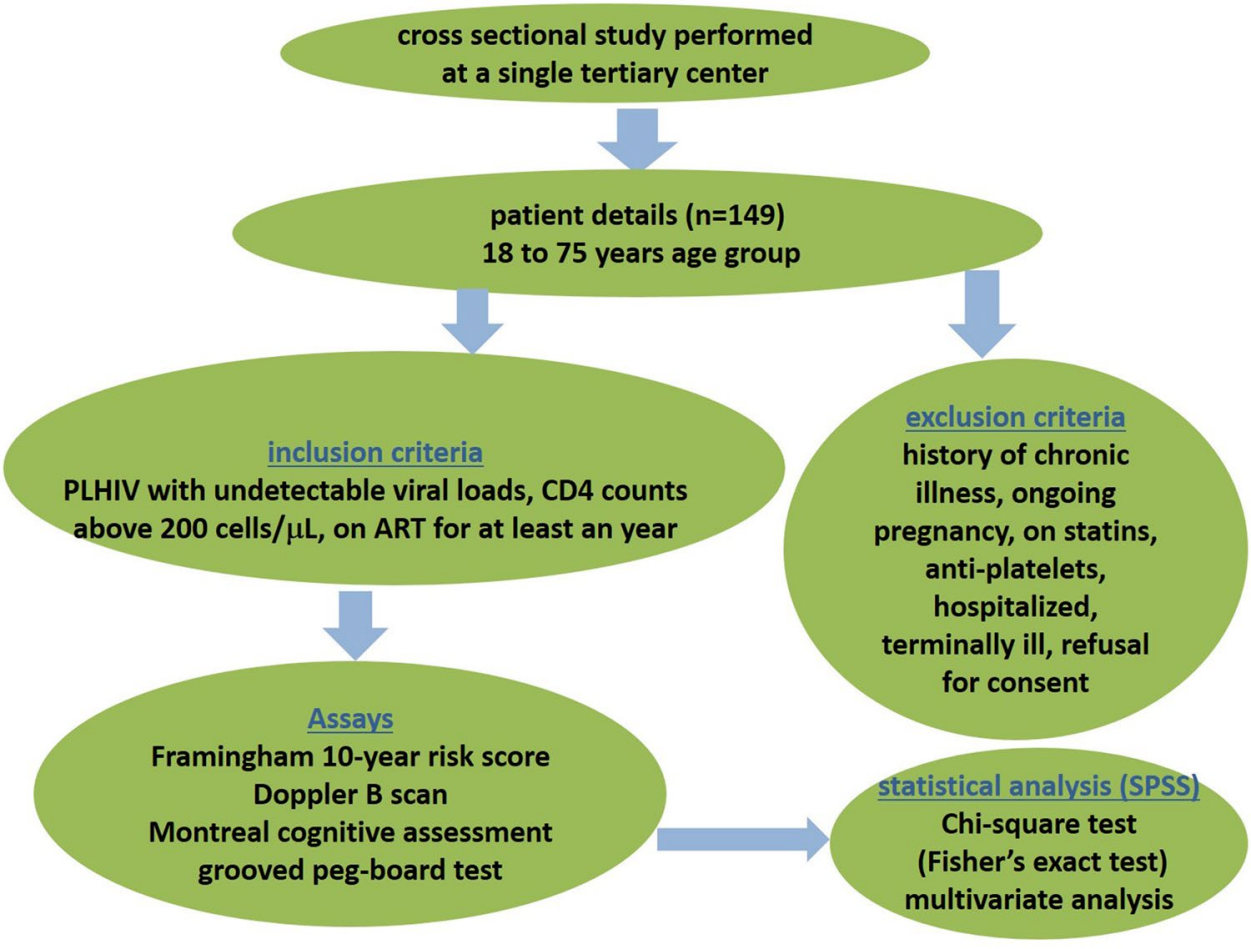
Schematic representation of the study design, patients’ details, inclusion and exclusion criteria, assays, and statistical analysis performed in this study

The study design, patients’ details, inclusion and exclusion criteria, assays, and statistical analysis performed in this research are schematically represented in Fig. 2.

## Results

### Population attributes

149 patients with undetectable viral load (< 20 HIV-1 RNA copies/mL) with a mean age of 47.98 ± 9.36 years participated in the research. The salient features of the population are briefed in Table 1. Table 2 represents the various anti-retroviral regimens and their durations, which participants received in this study. All patients were on cART, for a median 8 years (IQR 5–14 years). Diabetes, hypertension, and hypercholesterolemia were the most common comorbidities in the population. Among the participants, 93 (62.4%) had zero, 39 (26.2%) had one, and 14 (9.4%) had two comorbidities. Forty-nine participants (32.9%) had a history of opportunistic infections, including pulmonary and extrapulmonary tuberculosis, oral/oropharyngeal candidiasis and cytomegalovirus retinitis.

**Table 1:** Demographic characteristics of the study population (*n* = 149)

Characteristics	Mean ± SD/median (IQR)
Age (years)	47.98 ± 9.36
Male gender	86 (57.7%)
Body mass index (kg/m^2^)	22.76 ± 4.39
Education (years)	7.97 ± 3.81
Duration of HIV infection (years)	9 (5–15)
Duration of treatment (years)	8 (5–14)
CD4 count (cells/μL)	516 (392–690)
Past opportunistic infections	49 (32.9%)
Framingham 10-year risk	8% (4–12%)

**Table 2:** Anti-retroviral regimens and durations of the participants in this study

Regimen	Number (percentage)	Mean duration in years
TDF+3TC+EFV	45 (30.2)	5.2
TDF+3TC+DTG	46 (30.9)	10.1
AZT+3TC+NVP	21 (14.1)	12
TDF+3TC+ATV/r	21 (14.1)	12.5
AZT+3TC+ATV/r	5 (3.4)	6.4
Others	11 (7.4)	6.6

*3TC* lamivudine, *ATV/r* atazanavir/ritonavir, *AZT* zidovudine, *DTG* dolutegravir, *EFV* efavirenz, *NVP* nevirapine, *TDF* Tenofovir

### Carotid intima-media thickness in PLHIV on stable cART

While the carotid intima-media thickness varies with age and sex, ≥ 0.9 mm is the accepted cut-off, indicating the asymptomatic organ damage. This is per the guidelines of the ‘European Society of Cardiology/ European Society of Hypertension’ (Simova 2015).

The sidewise comparison of the cIMT of this study group is represented in Table 3. A higher proportion of individuals with CD4 count 200–349 cells/μL had abnormal cIMT when paralleled to those with CD4 counts 350–499 cells/μL and ≥ 500 cells/μL (72.4% vs 55% and 60% respectively), still this variance was not statistically significant (*p* = 0.328). Overall, 91 (61.1%) patients in this study showed cIMT ≥ 0.9 mm on one of the sides.

**Table 3:** Carotid intima-media thickness (cIMT) in the study population (*n* = 149)

cIMT	Mean ± SD	Median	IQR
Right side	0.965 ± 0.47	0.9	(0.7–1.15)
Left side	0.998 ± 0.53	0.9	(0.7–1.2)

Measurements are in mm

### Cardiovascular risk factors in PLHIV on stable cART

The cardiovascular risk factors prevalent in our study have been summarized in Table 4. 37.6% (*n* = 56) patients had none, 34.2% (*n* = 51) had one, 23.5% (*n* = 35) had two, and 4.7% (*n* = 7) had three cardiovascular risk factors correspondingly. The median cardiovascular risk factor was 1 (IQR 0–2). Hypercholesterolemia (30.9%) was the most common cardiovascular factor, followed by a high BMI of ≥ 25 kg/m^2^ (26.8%).

**Table 4:** Cardiovascular risk factors in the study population (*n* = 149)

Cardiovascular risk factors	*N* (%)
Diabetes	12 (8.1)
Hypertension	31 (20.8)
BMI ≥ 25 kg/m^2^	40 (26.8)
Current tobacco smoking	10 (6.7)
Hypercholesterolemia	46 (30.9)
Ischemic heart disease	5 (3.4)

### Association between cardiovascular risk factors, cIMT, and neuro-cognitive function

The average score in MoCA was 22.31 ± 3.77. Only 18.8% (*n* = 28) of the participants scored within the normal range of cognition. Mild cognitive impairment was present in 71.8% (*n* = 107) patients, moderate cognitive impairment in 9.4% (*n* = 14), and no participants had severe cognitive impairment. The association of cardiovascular risk factors, cIMT, and MoCA scores is summarized in Table 5.

**Table 5:** The association between cardiovascular risk factors and Montreal cognitive assessment scores (*n* = 149)

No cardiovascular impairment (*n* and %)	Mild cardiovascular impairment (*n* and %)	Moderate cardiovascular impairment (*n* and %)	‘*p*’ value (chi-square test)	Ordinal logistic regression, odds ratio (95% CI)	*χ*^2^	*p* value	No cardiovascular impairment (n and %)
Age (in years)							
≤ 30	3 (50%)	3 (50%)	0 (0%)				
31–50	18 (20.7%)	61 (70.1%)	8 (9.2%)	0.218			
51–70	7 (12.5%)	43 (76.8%)	6 (10.7%)				
Gender							
Male	14 (16.3%)	62 (72.1%)	10 (11.6%)	0.414			
Female	14 (22.2%)	45 (71.4%)	4 (6.3%)				
Diabetes mellitus							
Yes	27 (19.7%)	98 (71.5%)	12 (8.8%)	0.469			
No	1 (8.3%)	9 (75%)	2 (16.7%)				
Hypertension							
Yes	24 (20.3%)	86 (72.9%)	8 (6.8%)	0.084			
No	4 (12.9%)	21 (67.7%)	6 (19.4%)				
Serum cholesterol (mg/dl)							
< 200	20 (19.4%)	74 (71.8%)	9 (8.7%)	0.894			
≥ 200	8 (17.4%)	33 (71.7%)	5 (10.9%)				
HDL-C level (mg/dl)							
≥ 60	6 (50%)	6 (50%)	0 (0%)				
40–60	15 (16.1%)	67 (72%)	11 (11.8%)	0.045*	6.59* (1.65,26.27)*	10.41*	0.001**
< 40	7 (15.9%)	34 (77.3%)	3 (6.8%)		8.62* (2.33,31.89)*	7.131*	0.008*
Cigarette smoking							
Yes	3 (30%)	4 (40%)	3 (30%)	0.029*	2.02 (0.45,9.02)	0.847	0.357
No	25 (18%)	103 (74.1%)	11 (7.9%)				
Systolic blood pressure (mm Hg)							
< 120	9 (17.3%)	39 (75%)	4 (7.7%)				
120–139	17 (20.5%)	61 (73.5%)	5 (6%)	0.012*	0.63 (0.25,1.43)	1.221	0.269
≥ 140	2 (14.3%)	7 (50%)	5 (35.5%)		3.36 (0.88,12.89)	3.129	0.077
Framingham risk							
< 10%	24 (19.8%)	88 (72.7%)	9 (7.4%)	0.215			
≥ 10%	4 (14.3%)	19 (67.9%)	5 (17.9%)				
Body mass index (kg/m2)							
< 18.5	1 (4.2%)	19 (79.2%)	4 (16.7%)				
18.5–24.9	18 (21.2%)	61 (71.8%)	6 (7.1%)	0.246			
≥ 25	9 (22.5%)	27 (67.5%)	4 (10%)				
cIMT in mm							
Normal	12 (20.7%)	43 (74.1%)	3 (5.2%)	0.358			
Abnormal	16 (17.6%)	64 (70.3%)	11 (12.1%)				

Statistical significance *p < 0.05; **p < 0.001

Using the Chi-square test, a higher proportion of participants who had lower HDL-C levels (*p* = 0.045), were currently smoking (*p* = 0.029), had systolic blood pressure ≥ 140 mmHg (*p* = 0.012), and had education ≤ 12 years (more down than senior secondary education/ pre-university or its equivalent; *p* = 0.017) had poor cognitive performance. Other cardiovascular risk factors (as mentioned in Table 5), current use of protease inhibitors (*p* = 0.470), and CD4 cell counts (in cells/μL, *p* = 0.354) were not associated with impaired scores on MoCA.

On further ordinal logistic regression analysis for the significant variables, the odds of having cognitive impairment measured is 8.62 (95% CI 2.33–31.89) times higher for individuals with HDL-C < 40 mg/dl (Wald *χ*^2^(1) = 10.41, *p* = 0.001) and 6.59 (95% CI 1.65–26.27) times higher for individuals with HDL-C 40–60 mg/dl (Wald *χ*^2^(1) = 7.131, *p* = 0.008), when compared to those with HDL-C ≥ 60 mg/dl in each case. Individuals with education less than senior secondary level (≤ 12 years) have 6.39 (95% CI 2.19–18.69) higher odds of impaired cognition on MoCA when compared to those who have attained higher than senior secondary level (> 12 years) of education, Wald *χ*^2^(1) = 11.51, *p* < 0.001 (Table 5). Average time taken to complete the Grooved peg board was 75.06 ± 19.25 s with the dominant hand and 80.22 ± 19.91 s using the non-dominant hand. 11 (7.4%) and 14 (9.4%) participants were classified as having mild cognitive impairment with the dominant and non-dominant hand, respectively.

Impaired performance on the grooved pegboard test with the dominant hand is associated with elevated systolic blood pressure. 21.4% of those with systolic blood pressure ≥ 140 mmHg had impairment on the grooved pegboard test compared to groups with < 120 mmHg and 120–139 mmHg (11.5% and 2.4%, respectively, p = 0.015). Pathological performance on the grooved pegboard test with a non-dominant hand showed no association with cardiovascular risk factors. CD4 count (dominant hand: *p* = 0.311; non-dominant hand: *p* = 0.429), current use of protease inhibitor regimen (Fisher’s exact test: dominant hand, *p* = 0.460; non-dominant extremity, *p* = 0.735), and level of education (Fisher’s exact test: dominant, *p* = 0.168; non-dominant, *p* = 0.404) were not associated with impaired performance on grooved pegboard test. Abnormal cIMT (≥ 0.9 mm on any one side) is associated with a greater Framingham 10-year risk percentage and male sex (Table 6). On binary logistic regression, neither of the two [sex: OR = 1.79, 95% CI (0.89, 3.61), *p* = 0.104; Framingham risk %: OR = 2.21, 95% CI (0.80, 6.08), *p* = 0.124] remained statistically significant. Other cardiovascular risk factors, current use of protease inhibitors (*p* = 0.978), and CD4 cell count (in cells/μL, *p* = 0.328) were not associated with increased cIMT. We could not evaluate the ischemic heart disease with the other variables because of the smaller samples in the population (*n* = 5).

**Table 6:** Association between cardiovascular risk factors, cIMT, and grooved peg board test scores (*n* = 149)

Patient profile	Carotid intima-media thickness		Impairment as per the grooved pegboard test *n* (%)		*n* (%)	
	≥ 0.9μμ ν (%)	*p* value	Dominant hand	*p* value	Non-dominant hand	*p* value
Age (in years)						
≤ 30	3 (50%)		0 (0%)		0 (0%)	
31–50	47 (54%)	0.061	5 (5.7%)	0.422	6 (6.9%)	0.243
51–70	41 (73.2%)		6 (10.7%)		8 (14.3%)	
Gender						
Male	59 (68.6%)	0.028*	6 (7%)	1.000^#^	8 (9.3%)	0.963
Female	32 (50.8%)		5 (7.9%)		6 (9.5%)	
Diabetes mellitus						
Yes	8 (66.7%)	0.679	2 (16.7%)	0.217^#^	2 (16.7%)	0.314^#^
No	83 (60.6%)		9 (6.6%)		12 (8.8%)	
Hypertension						
Yes	23 (74.2%)	0.092	3 (9.7%)	0.699^#^	3 (9.7%)	1.000^#^
No	68 (57.6%)		8 (6.8%)		11 (9.3%)	
Body mass index (kg/m^2^)						
< 18.5	13 (54.2%)		3 (12.5%)		5 (20.8%)	
18.5–24.9	54 (63.5%)	0.699	7 (8.2%)	0.301	8 (9.4%)	0.052
≥ 25	24 (60%)		1 (2.5%)		1 (2.5%)	
Cigarette smoking						
Yes	8 (80%)	0.317^#^	1 (10%)	0.547^#^	1 (10%)	1.000^#^
No	83 (59.7%)		10 (7.2%)		13 (9.4%)	
Serum cholesterol (mg/dl)						
< 200	59 (57.3%)	0.155	9 (8.7%)	0.504^#^	11 (10.7%)	0.551^#^
≥ 200	32 (69.9%)		2 (4.3%)		3 (6.5%)	
HDL-C level (mg/dl)						
≥ 60	9 (75%)		2 (4.5%)		3 (6.8%)	0.327
40–60	57 (61.3%)	0.518	9 (9.7%)	0.334	11 (11.8%)	
< 40	25 (56.8%)		0 (0%)		0 (0%)	
Systolic blood pressure (mm Hg)						
< 120	32 (61.5%)		6 (11.5%)		6 (11.5%)	
120–139	49 (59%)	0.667	2 (2.4%)	0.015*	5 (6%)	0.152
≥ 140	10 (71.4%)		3 (21.4%)		3 (21.4%)	
Framingham 10-year risk						
< 10%	69 (57%)	0.035*	7 (5.8%)	0.220^#^	9 (7.4%)	0.089
≥ 10%	22 (78.6%)		4 (14.3%)		5 (17.9%)	
cIMT (in mm)						
Normal	58 (38.9%)		3 (5.2%)	0.530^#^	4 (6.9%)	0.404
Abnormal	91 (61.1%)		8 (8.8%)		10 (11%)	

Statistical significance *p < 0.05

^#^Fischer’s exact test value

## Discussion

The median cIMT in our study was 0.9 mm on either side and the mean was 0.965 ± 0.47 mm over the right side and 0.998 ± 0.53 mm over the left side. This value is higher than Sharma et al. (2018), where the mean cIMT measured 0.52 ± 0.084 mm and 0.51 ± 0.095 mm on the right and left sides. This difference may be because they had a lower mean age of their population compared to our study. The median cIMT was 0.68 and 0.70 mm (right and left sides) in a multicentric Italian cohort of comparable age and higher cardiovascular risks (Fabbiani et al. 2013). The cIMT measured in our study population is higher than previous studies. The difference may be because of the variability in the study design, as ours is a single-center study, which limits the generalization of results. The sample size was also small and is a cross-sectional study, which affects the interpretation of results. A cohort study will better compare the cIMT with the other studies. The higher cIMT in PLHIV which is observed in this study, supports the opinion of Fabbiani et al. (2013). They opined that PLHIV are known to have higher rates of cardiovascular risks. The higher cIMT dictates the damage caused by coronary artery disease risk factors on the arterial wall (Gnasso et al. 1996). It was reported that cIMT higher than 1.15 mm has an increased risk of having coronary artery disease and the stenosis of aortic arch arteries (Kablak-Ziembicka et al. 2004). This present study observed the prevalence of associated cardiovascular risk factors almost similar to the other Indian study by Gupta and Venugopal (2020), in which the frequency was 58.9%. In our population, 62% PLHIV had at least one cardiovascular risk vs. 91.4% in an Italian cohort. The higher prevalence in the Italian population may be because of the demographic characteristics, ancestral variations, and lifestyle changes as Italy is a developed nation. In comparison to previous reports, the prevalence of lipid profile abnormality (30.9% vs. 66% and 61.2%), current smoking (6.7% vs. 50% and 54%), and high Framingham 10-year risk (14%) in our study had ≥ 10% risk vs. 21% (≥ 10% risk) and 22% (> 10% risk)] were lower in our study while the prevalence of diabetes (8.1% vs. 6% and 8.2%) and hypertension (20.8 vs. 15.1%) were comparable (Ciccarelli et al. 2015; Fabbiani et al. 2013). Higher total cholesterol levels, BMI, and blood pressure are risk factors associated with increased cardiovascular event rates (Bijker et al. 2019). However, these are modifiable risk factors and appropriate interventions help reduce the likelihood of patients suffering from subsequent cardiovascular events. In our study, pathological cIMT was significant in males and had a high Framingham risk score (≥ 10% in 10 years). Interestingly, no other cardiovascular risk factor was connected with increased cIMT in our study, which does not agree with the previous studies (Jericó et al. 2006; Monsuez et al. 2009). Male sex, a traditional cardiovascular risk factor, is expected to be associated with increased cIMT. Previously, Jericó et al. (2006) conducted a study in PLHIV, which identified the subclinical atherosclerosis (cIMT > 0.8 and presence of plaque) to be linked to the other cardiovascular risk factors, Framingham 10-year risk ≥ 10% as well as those receiving cART. Based on our results, we opine that these risk factors intricately operate to cause subclinical atherosclerosis in PLHIV.

Former studies have found a significant association between cIMT and cognitive function in PLHIV (Ciccarelli et al. 2015; Fabbiani et al. 2013), while few have not (Hiransuthikul et al. 2019). In our study, no association between cIMT and MoCA scores or the grooved pegboard test was observed, suggesting that cIMT may have limited utility in screening for neurocognitive dysfunction in PLHIV. We observed no association between cIMT with the current use of protease-inhibitor drugs. Exposure to the same was found to be accompanied by increased cIMT in former reports (Jericó et al. 2006). However, the previous exposure was not elicited in our study. Increased cIMT suggests subclinical atherosclerosis and is connected with the bigger hazard of cardiovascular events like stroke (Saleh 2010). In this study, 81.2% of the patients have cognitive impairment as assessed by MoCA. The prevalence in this research is higher than previously reported, ranging from 36% to 71.1% (Fabbiani et al. 2013; Cook et al. 2016; Gupta and Venugopal 2020; Ganapathy et al. 2021). This could be partly attributed to the varied tools used for measuring the cognitive functions. No severe cognitive impairment was identified in this population using MoCA reflecting the trend of prevalence- mainly driven by milder forms of impairment than severe ones (Heaton et al. 2010). The factors linked to cognitive function in our study are HDL-C, smoking, high systolic blood pressure, and level of education. Using multivariate analysis, only lower HDL-C and education less than senior secondary level were independently associated with poor cognitive performance.

The study by Crichton et al. (2014) observed an association of higher HDL-C levels with better cognitive outcomes in patients aged between 60 and 90 years. However, two other studies, which analyzed the same in PLHIV described no association of these higher HDL-C levels (McCutchan et al. 2012; Schouten et al. 2016). This is the first study in PLHIV, which describes the association between the low HDL-C level and poorer cognitive outcomes. The function of HDL-C in cardiovascular health and cerebrovascular systems may explain the cognitive impairment in these patients. Atherosclerosis of carotid arteries and cerebral vessels will lead to circulatory disturbances and chronic hypoperfusion with subsequent impaired cognitive function. HDL cholesterol facilitates the reverse cholesterol transport from the peripheral cells to the liver, thereby guarding the arteries against atherosclerosis, improving cognitive function. In addition, HDL-C performs anti-inflammatory, antithrombotic, antioxidant, and antiapoptotic functions. In virally suppressed individuals, HDL-C levels will be reduced and HDL subclass distribution shifts, impeding reverse cholesterol transport. HIV infection, being a state of chronic inflammation, diminishes the role of HDL-C’s anti-inflammatory action (Hudson et al. 2020). However, the exact position of HDL cholesterol in cognitive impairment is not well established. While there is a lack of a well-defined pharmaco-therapeutic approach to counter the elevated cardiovascular risks in PLHIV, the administration of rosuvastatin has been shown to reduce the mean level of non-HDL cholesterol after 12 and 24 months of treatment. The mean values of cIMT decreased after 24 months in the extra cranial carotid arterial system compared to the baseline values, which was associated with good tolerability profile (Calza et al. 2013).

Considering the observed associations between cardiovascular risk factors, cIMT, and cognitive function, guidelines should be available about the clinical management and interventions aimed at reducing cardiovascular risk and improving the cognitive outcomes in people living with HIV. Statins and lifestyle modifications are advised to manage the dyslipidemia in PLHIV, which can lead to coronary artery disease. Smoking cessation should be done in smokers. Current guidelines mainly focus on prescribing the accurate intensity of statin therapy to decrease LDL-C levels (Stein 2016).

Cognitive screening tools, modified HIV dementia scale, and mini-mental status examination can be tried in dementia of PLHIV. Montreal cognitive assessment can evaluate the multiple cognitive domains. MRI brain can be taken to rule out any reversible pathological changes (Seitz et al. 2007). Psychostimulants can be prescribed to treat symptoms like fatigue, cognitive impairment, and depression in PLHIV. The delirium in PLHIV is managed in a similar way as in the non-HIV infected. The underlying and contributing factors should be taken care of along with reassurance and counseling. They can be started with a lower dose of haloperidol as an antipsychotic medication (Seitz et al. 2007). It was reported that chlorpromazine, risperidone, quetiapine, and olanzapine are helpful in PLHIV (Watkins and Treisman 2015). Since the adverse effects are high in PLHIV, the antidepressant medications should be initiated at the sub-therapeutic dose, and the dose can be increased slowly if required. Tricyclic antidepressants help these patients (Seitz et al. 2007). Clomipramine and imipramine have anti-inflammatory and neuroprotective roles by regulating the glial cells (Harezlak et al. 2011).

Longitudinal studies in PLHIV are needed to identify specific domains impaired with HDL cholesterol levels. Current smoking was associated with poor cognitive performance on univariate analysis but not significant by logistic regression. Literature suggests that PLHIV smokers experienced early deterioration in psychomotor speed. Smoking was concomitant with cognitive waning in non-PLHIV, especially in men (Fabbiani et al. 2013; Sabia et al. 2012). In a recent study, poor neuropsychological assessment and grooved pegboard test performance was independently associated with high systolic blood pressure (Goldstein et al. 2017). Lower educational achievement in PLHIV, less than a bachelor’s degree in a Thailand-based study (Hiransuthikul et al. 2019), and less than 10 years in an Indian research were independently associated (Gupta and Venugopal 2020) with lower cognition. However, the role played by education in HAND is still being determined. Gupta and Venugopal (2020) reported that the presence of one or more cardiovascular risk factors in PLHIV was linked to cognitive impairment. However, data on specific cardiovascular risks were lacking. In studies across the world, Diabetes (McCutchan et al. 2012; Schouten et al. 2016), hyperlipidemia, high BMI (McCutchan et al. 2012; Ciccarelli et al. 2015), hypertension, tobacco use (Fabbiani et al. 2013), and high waist-hip ratio (Schouten et al. 2016) were independently linked to unfavorable global cognitive score. Other cardiovascular risk factors or demographic characteristics were not independently linked to cognitive impairment in our study. The association of poor performance with overall cognitive assessment and grooved pegboard test with higher systolic blood pressure concur with previous results (Waldstein et al. 2005).

Vascular circulatory derangement is one of the mechanisms of cognitive impairment in PLHIV. Earlier HIV invasion of the brain and inflammatory response to low-level persistent viremia inadequately controlled by cART are significant in the pathogenesis of HAND. Psychiatric co-morbidities such as depression in PLHIV also play a role in poor cognitive function (Cook et al. 2016; Hiransuthikul et al. 2019). Some participants were administered cART when CD4 counts were used to guide therapy initiation. Treatment with cART in the early stages of HIV infection, regardless of CD4 counts, is the standard of care presently. It is expected to play a part in limiting cognitive impairment mainly to its milder forms. However, further research in this regard is necessary since the current literature reports a favorable role of cART on neurocognitive function limited to older PLHIV with poor health and advanced immunosuppression (Nakasujja et al. 2010). Cognitive deficits are significant when they affect daily functioning in PLHIV, thereby quality of life and treatment compliance (Gupta and Venugopal 2020). PLHIV (CD4 counts > 400 cells/μL) suffered from lower quality of life than matched controls, significant fatigue, and poor physical and social functioning (Cook et al. 2016).

A study from Indian samples observed that, cardiovascular diseases in PLHIV if diagnosed at a younger age, can have significant morbidity risk from them. It is suggested that performing proper screening and management with statins is considered an important strategy to prevent the morbidity (Marbaniang et al. 2017). Another study from our local population observed that more than 50% of the PLHIV were associated with moderate or high risk for cardiovascular diseases in the 5 years post-exposure period (Mallya et al. 2020). It was opined that cultural lifestyle modifications need to be done in PLHIV in our sample population. Early diagnosis and best management can decrease the mortality due to cardiovascular diseases in PLHIV (Mallya et al. 2020). In this clinical research, we have attempted to measure the cIMT, cardiovascular risk issues, and its association with cognitive impairment. There are few studies globally available about this subject. However, the same are not available in the Indian sample population. Through this study, the authors have attempted to explore the associated risks for cognitive impairment among PLHIV in this region.

The limitations of the study need to be deliberated before interpreting the results. The study included a total of 149 patients, which is a smaller sample size and may limit the generalizability of the findings. A larger sample size would have provided more robust results. This present research is a cross-sectional model, which provides data from the study period alone and is prone to uncontrolled biases. The cross-sectional design can only provide a snapshot of the data at a specific point in time. Longitudinal studies are needed to establish causal relationships and determine changes over time. The absence of controls in this study predisposes to overemphasis or underestimation of some results due to a lack of comparison between HIV and non-HIV groups. Some of the data collected in our study, such as comorbidities and cardiovascular risk factors, relied on self-reporting by the participants. Self-reporting might have been subjected to recall bias or misinterpretation, which may affect the accuracy of the results in this study. The study was conducted at a single center, which may limit the representativeness of the findings to a broader population. The results from a single center may not apply to other settings or populations. This limits the generalization of results in comparison with a multi-center study. Furthermore, a cohort study can reveal long-term outcomes of cognitive impairment with cardiovascular risks and cIMT. The outcomes of the grooved pegboard test may not be reliable due to skewed data distribution (only 7.4% and 9.4% were impaired with dominant and non-dominant hands, respectively). We used the MoCA, a standard concise tool that allows healthcare professionals a faster diagnosis of cognitive impairment and patient care than a robust method for domain-specific neurocognitive assessment. The details about the specific cognitive domains assessed and the interpretation of MoCA scores to determine the impairment levels were as per Nasreddine et al. (2005). The cut-off for grading the mild, moderate, and severe cognitive impairment were incorporated from the official website of the MoCA tool. While the MoCA test is a widely used tool, it may not have captured the full spectrum of cognitive impairment. Additional neuropsychological tests could have provided a more comprehensive assessment. Using comprehensive assessment methods in further studies can provide details of the nature of the neurocognitive impairment. This study did not cover the assessment of specific domains of cognition and their association with cardiovascular risk factors. This study’s evaluation of ischemic heart disease is limited because of the small number of samples. This limitation restricts understanding the relationship between cardiovascular risk factors and ischemic heart disease in PLHIV. The cardiovascular risk factors or cognitive function based on the specific medications or drug classes used in this study are also not considered for the analysis. The study did not account for potential confounding factors that may have influenced the outcomes. This study has not assessed other factors affecting cardiovascular risk or cognitive impairment in PLHIV, such as substance abuse, mental health conditions, socioeconomic conditions, medication use, and lifestyle behaviors. We have not attempted to assess the quality of life or impaired functioning as a part of this study, and there is potential for further research in the Indian HIV population.

Using comprehensive assessment methods in further studies can provide details of the nature of the neurocognitive impairment. Further research could be directed at evaluating their role in cognitive functions and specific management. Further exploration of the relationship between cardiovascular risk, cIMT, and cognitive impairment must be done in PLHIV. Future research is essential to formulate the guidelines and preventive measures to delay the onset of neurological involvement and decrease the incidence of neurocognitive disorders. The regression formula and the correlation matrix for the association between cardiovascular risk factors, cIMT, and neurocognitive function can be formulated. The future implication of this research also includes studying the microbiota in our sample population, infected with HIV.

## Conclusion

The present study observed a high prevalence of elevated cIMT, cardiovascular diseases, and mild and moderate cognitive deficiency in PLHIV on stable cART. It was evident that the measurement of cIMT has a minimal role in predicting cognitive impairment. The cardiovascular risk factors, particularly the high HDL-C level and education level (senior secondary or lower), had higher odds of cognitive impairment. However, routine assessment of neuropsychological function using the appropriate battery of tests and managing modifiable risks is necessary to avoid future cardiovascular events.

## Abbreviations

3TC: Lamivudine
ATV/r: Atazanavir/ritonavir
AZT: Zidovudine
cART: Combination anti-retroviral therapy
cIMT: Carotid Intima media thickness
DTG: Dolutegravir
EFV: Efavirenz
HAND: HIV-associated neurocognitive disorder
HDL-C: High-density lipoprotein cholesterol
HIV: Human immunodeficiency virus
HIV-1 RNA: Human immunodeficiency virus-1 ribonucleic acid
MoCA: Montreal cognitive assessment
NVP: Nevirapine
PLHIV: People living with HIV
TDF: Tenofovir
